# An Ultrafast Active Quenching Active Reset Circuit with 50% SPAD Afterpulsing Reduction in a 28 nm FD-SOI CMOS Technology Using Body Biasing Technique

**DOI:** 10.3390/s21124014

**Published:** 2021-06-10

**Authors:** Mohammadreza Dolatpoor Lakeh, Jean-Baptiste Kammerer, Enagnon Aguénounon, Dylan Issartel, Jean-Baptiste Schell, Sven Rink, Andreia Cathelin, Francis Calmon, Wilfried Uhring

**Affiliations:** 1 ICube Research Institute, University of Strasbourg and CNRS, 23 Rue du Loess, CEDEX, 67037 Strasbourg, France; dolatpoorlakeh@unistra.fr (M.D.L.); jb.kammerer@unistra.fr (J.-B.K.); faguenounon@unistra.fr (E.A.); jbschell@unistra.fr (J.-B.S.); sven.rink@etu.unistra.fr (S.R.); 2 Univ Lyon, INSA Lyon, CNRS, INL, UMR5270, 69100 Villeurbanne, France; dylan.issartel@insa-lyon.fr (D.I.); francis.calmon@insa-lyon.fr (F.C.); 3 STMicroelectronics, 38920 Crolles, France; andreia.cathelin@st.com

**Keywords:** afterpulsing, avalanche charge, active quenching, avalanche detection, inverter, body biasing, Fully Depleted Silicon On Insulator (FD-SOI), Single Photon Avalanche Diode (SPAD)

## Abstract

An ultrafast Active Quenching—Active Reset (AQAR) circuit is presented for the afterpulsing reduction in a Single Photon Avalanche Diode (SPAD). The proposed circuit is designed in a 28 nm Fully Depleted Silicon On Insulator (FD-SOI) CMOS technology. By exploiting the body biasing technique, the avalanche is detected very quickly and, consequently, is quenched very fast. The fast quenching decreases the avalanche charges, therefore resulting in the afterpulsing reduction. Both post-layout and experimental results are presented and are highly in accordance with each other. It is shown that the proposed AQAR circuit is able to detect the avalanche in less than 40 ps and reduce the avalanche charge and the afterpulsing up to 50%.

## 1. Introduction

Regarding their maximum sensitivity in the optical signal detection and very high temporal resolution in the range of tens of picoseconds, Single Photon Avalanche Diodes (SPADs) play a significant role in a variety of applications that require photon counting or photon timing measurements. Light Detection and Ranging (LiDAR), quantum computing, quantum cryptography, Fluorescence Lifetime Imaging Microscopy (FLIM), and Time-Correlated Single Photon Counting (TCSPC) are some of these applications [1,2,3].

A SPAD is a diode, biased reversely up to an excessive voltage (excess bias voltage (V_ex_)) over its breakdown voltage. In this state of operation, which is called the Geiger mode, the electrical field across the diode junction is extremely high. Therefore, a free carrier—either a photo-generated one or even a non-photo-generated one—can trigger a positive feedback loop of impact ionization that results in an avalanche of carriers, the avalanche current. After it starts, the avalanche must be quenched to prevent the damage of the SPAD. The quenching process can simply be done by a resistor, which is called the Passive Quenching (PQ) or by means of active circuits, called the Active Quenching (AQ). In both methods, the carrier flow inside the Space Charge Region (SCR) of the SPAD is stopped by lowering the voltage over the SPAD (biasing it out of the Geiger mode) in order to reduce the electrical field and, thus, to stop the impact ionization process [4,5].

During the passing of the avalanche current through the SCR, some of the carriers are captured by the trapping centers of the semiconductor. These trapping centers dismiss the trapped carriers after a time that can take up to several microseconds, depending on the temperature and the type of the trap. If the release happens when the SPAD is out of the Geiger mode and not yet reset to its initial condition, the unleashed carrier cannot trigger the impact ionization process. Indeed, when the voltage over the SPAD is below its breakdown voltage, the SPAD is unable to generate an avalanche. This is why this duration is called the dead time or the hold-off time. However, if the carrier is freed when the SPAD, after the quenching, is reset to the Geiger mode operation, the released carrier can initiate an avalanche which results to a false photon detection. This spurious avalanche is called the afterpulse, and the aforementioned phenomenon is called the afterpulsing effect. The afterpulsing effect is a correlated noise since it is a consequence of a previous main photo-generated avalanche. It strongly depends on the number of the trapping centers inside the semiconductor lattice and the number of the carriers inside the SAPD SCR. Afterpulsing, as well as the Dark Count Rate (DCR), are two main noise sources that deteriorate the signal-to-noise ratio of the SPAD [6,7,8]. 

Regarding the characteristics of the afterpulsing effect, there are a few ways to reduce it. Increasing the dead time sufficiently, allows the trapping centers to release the carriers without generating an avalanche. The dead time can be controlled by an active circuit (Active Reset (AR)) or by a resistor (Passive Reset). The big drawback of this method is a drastic reduction in the maximum photon count rate. The dynamic range of the SPAD is defined as the ratio of maximum count rate to the DCR and is a crucial characteristic in photon counting applications. Therefore, increasing the dead time is not a universal solution for afterpulsing reduction [9,10]. In addition, it should be noted that a common method to reduce the DCR is cooling down the SPAD, but, simultaneously, this prolongs the trapping time, so even a longer dead time is needed for the SPAD to get rid of the trapped carriers [7,11].

**Figure 1:**
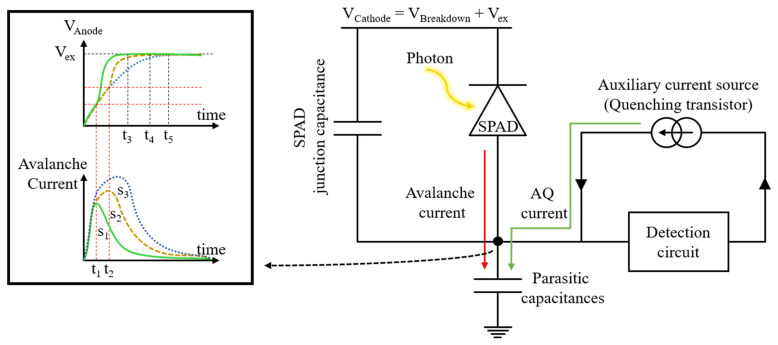
Symbolic block diagram of the afterpulsing reduction method by means of the AQ circuit.

From the viewpoint of a circuit designer, the only remaining solution to reduce the afterpulsing effect is reducing the number of avalanche charge carriers. Since the traps are usually too numerous to be saturated, therefore, the afterpulsing effect is proportional to the number of carriers inside the SCR [12,13]. Reducing the avalanche charge can reduce the afterpulsing without affecting the dynamic range due to the dead time extension. This is what an AQ circuit is expected to do [14]. An AQ circuit is mainly composed of a detection circuit which senses the avalanche and drives the other part of the circuit which is the quenching transistor. The quenching transistor lowers the voltage across the SPAD by discharging the junction capacitance of the SPAD and charging or discharging (depending on the configuration of the SPAD) all the parasitic capacitances at the dynamic node of the SPAD; see Figure 1.

The efficiency of the afterpulsing reduction based on the avalanche charge reduction strongly depends on the speed of the detection circuit in sensing the avalanche. A faster avalanche detection leads to a faster intervention by the quenching transistor which potentially results in a faster quenching that means less avalanche charge [15,16]. Most of the reported AQ circuits in the literature suffer from the slow detection circuit or do not clearly report an effective reduction of the afterpulsing effect.

In this paper, we present an Active Quenching—Active Reset (AQAR) circuit in a 28 nm Fully Depleted Silicon on Insulator (FD-SOI) CMOS technology. The AQAR system benefits from a very fast detection circuit, thanks to the body biasing feature of the FD-SOI technology. We will show that a faster avalanche detection results in a greater reduction of the afterpulsing effect.

The rest of the paper is organized as follows: In Section 2, a technique to design a very fast detection circuit is presented. In Section 3, the full schematic of the proposed circuit and its operation is described. Simulation and experimental results are given in Section 4, and Section 5 concludes the paper.

## 2. Fast Avalanche Detection

**Figure 2:**
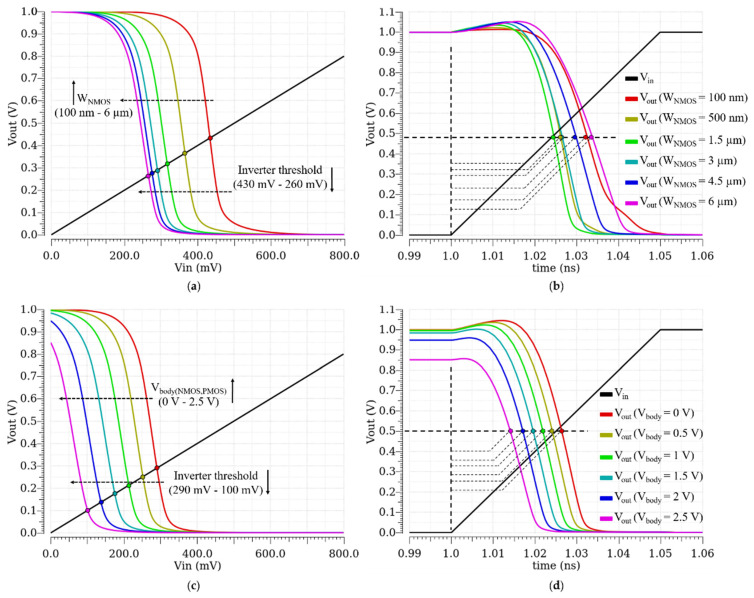
Voltage Transfer Characteristic (VTC) and transient behavior of an inverter: (**a**) VTC of an inverter for different NMOS widths; (**b**) transient response of an inverter for different NMOS widths; (**c**) VTC of an inverter for different body voltages of the NMOS and the PMOS; (**d**) transient response of an inverter for different body voltages of the NMOS and the PMOS.

Due to their design simplicity, small silicon area and low power consumption, inverters are popular nominees for the avalanche detection [17]. In order to turn an inverter to a fast detection circuit, it is needed to reduce the switching threshold voltage of the inverter. This enables the inverter to commute its output for very small voltage variations at its input. Generally, the switching threshold voltage reduction in the inverter is performed by a proper sizing of its transistors. In Figure 2a, it is shown how increasing the width of the NMOS reduces the switching threshold of the inverter. However, the threshold reduction in this method increases the parasitic capacitances due to the enlargement of the NMOS and, thus, increases the switching delay (the time interval between the beginning of the input signal and when the output signal reaches to half of its maximum swing) of the inverter, as shown in the transient response of the inverter in Figure 2b.

**Figure 3:**
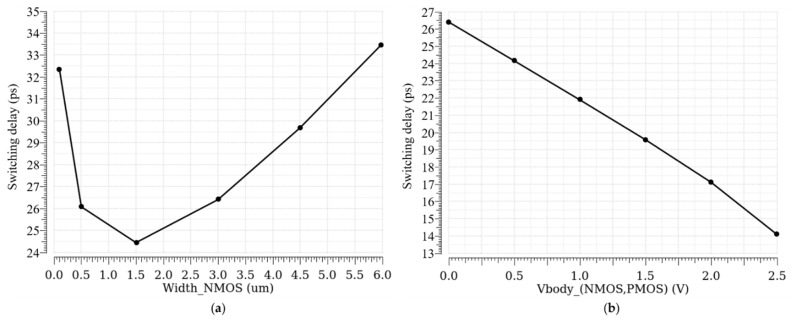
Switching delay of an inverter versus: (**a**) NMOS width; (**b**) body voltage of the NMOS and the PMOS.

Figure 3a shows the switching delay of the inverter when the width of its NMOS is increasing. Even though, at the beginning, increasing the width of the NMOS reduces the switching delay, however, after a certain optimum width, the switching delay starts to increase and slows down the inverter. The threshold is well reduced, as shown in Figure 2a, but the overall performance of the detection is worsened, and the switching delay becomes longer than 24 ps in the used technology.

There is another technique that can reduce the switching threshold of an inverter without affecting its parasitic capacitances. Indeed, if the threshold voltage of the NMOS in the inverter is reduced, and/or the threshold voltage of the PMOS is increased (in absolute value), the switching threshold reduces. By means of the body biasing, it is possible to change the threshold voltage of a MOSFET without increasing its parasitic capacitances [8]. Figure 2c,d show how body voltage variation can reduce the switching threshold of the inverter without deteriorating its transient behavior. In addition, Figure 3b illustrates the linear relationship between the switching delay of the inverter and the body voltage of its MOSFETs, which can be as short as 14 ps for a given size of the transistors.

**Figure 4:**
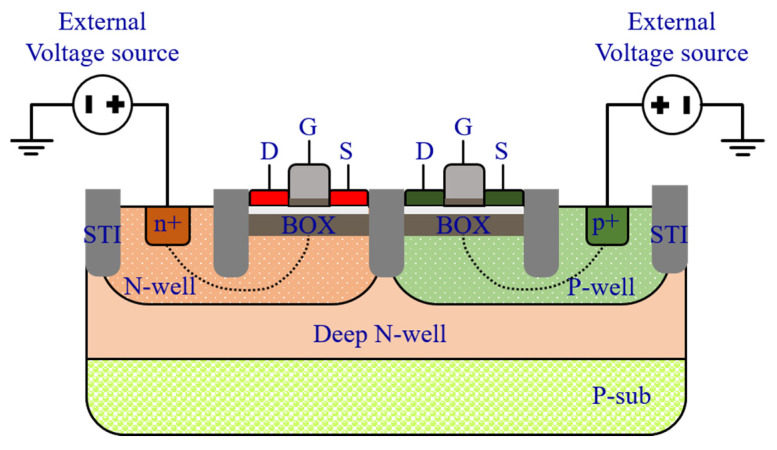
A symbolic cross section of the FD-SOI CMOS technology with the flip well MOSFETs and the body biasing illustration.

But how this body biasing technique is performed? In order to effectively reduce the switching threshold of the inverter, it is required to apply enough voltage to the bodies of its transistors. However, in the regular bulk CMOS technology, the body voltage is limited by the leakage current due to the forward bias of the parasitic diodes. In Figure 4, a symbolic cross section of the FD-SOI CMOS technology is shown. The MOSFETs are fabricated over an ultra-thin buried oxide layer (25 nm), and their channel (7 nm) is fully depleted. The technology is also called Ultra-Thin Body and Box (UTBB) FD-SOI. The buried oxide layer isolates the transistor from their well, therefore extremely reduces the leakage current. Therefore, the two parasitic diodes between the Drain and the Well and between the Source and the Well are eliminated [18]. In addition, it should be mentioned that the family of the MOSFETs used in this paper is one of flip well transistors, in which the PMOS is fabricated in the P-Well, and the NMOS is fabricated in the N-Well, to obtain a lower threshold voltage through the forward body biasing. The buried oxide layer is potentially a second Gate (back Gate), besides the conventional Gate (front Gate). Thus, as it is shown in Figure 4, it is possible to directly connect a voltage source to it and modulate the threshold voltage of the MOSFET. The body biasing factor (the ratio between the threshold voltage and the body voltage) in this technology is about 85 mV/V. So, theoretically, it is possible to reduce the switching threshold of the inverter for about 255 mV, by only the body biasing. Finally, by combining the two aforementioned methods, the body biasing and a proper sizing, it is possible to design an inverter with an ultra-low switching threshold and very short switching delay.

## 3. The AQAR Circuit Based on the Body Biased Inverter

**Figure 5:**
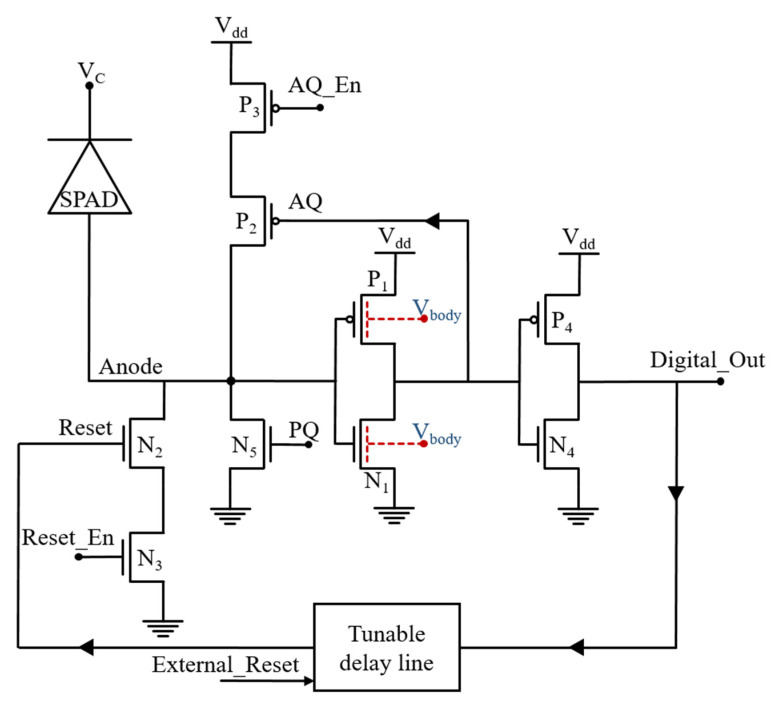
Schematic of the proposed body biased, inverter base AQAR circuit.

An AQAR circuit based on the ultra-low switching threshold inverter is presented in Figure 5. The detection inverter is implemented with P_1_ and N_1_ MOSFETs. P_1_ has the minimal width and length (W/L = 80 nm/30 nm), and, for N_1_, W/L = 3 µm/30 nm. Their bodies are connected to an external voltage source that provides the body biasing. For a constant body voltage, this sizing results in an optimal switching delay, and, by modulating the body voltage, it is possible to reduces more this optimal switching delay.

When an avalanche happens, the anode voltage starts to rise. Thanks to the ultra-sensitivity of the detection inverter, it can detect the avalanche at its very beginning. Then, the output of the detection inverter commutes and drives the AQ transistor P_2_. The P_2_ conducts and contributes to the quenching process and makes it faster by charging the anode node. The detection and the quenching time can be controlled externally through the body voltage modulation.

The output of the detection inverter also drives a second inverter (P_4_, N_4_), which generates a digital signal for the readout electronics and also drives a tunable delay line. The delay line regulates the dead time by controlling the AR transistor N_2_. It can provide a dead time of 5 ns, up to more than 50 ns.

P_3_ and N_3_ are serial switches for AQ and AR transistors, respectively. They are 10 times bigger than their corresponding AQ and AR MOSFETs, and they are used to enable or disable the AQ and the AR for measurement purposes. N_5_ is used as a tunable resistor for PQ operation. Its nominal gate voltage is 600 mV, which, with W/L = 400 nm/2 µm, yields an R_on_ of about 200 kΩ. When P_3_ is turned off, it allows PQ operation, which is used as the reference to investigate the effectiveness of the AQ circuit in the avalanche charge reduction.

## 4. Results

In this section, post-layout simulation results and experimental results are presented and discussed. It is shown that the experimental results are in accordance with the simulation results. The goal of the experiments is to investigate the effectiveness of the proposed AQAR circuit in the afterpulsing reduction with completely electrical measurements. 

### 4.1. Post Layout Simulation Results

The proposed body biased inverter base AQAR circuit is designed and laid out in a 28 nm FD-SOI CMOS technology. In order to simulate the behavior of the SPAD, a SPICE macro model is used [19]. The simulated SPAD is set to have a 100 fF junction capacitance and a serial resistance of 1 kΩ. The V_ex_ is set to 1 V, which is equal to the supply voltage in the used technology. Thanks to the P_3_ switch and N_5_, a comparison is made between PQ and AQ to see how much the AQ circuit can reduce the avalanche charge. 

**Figure 6:**
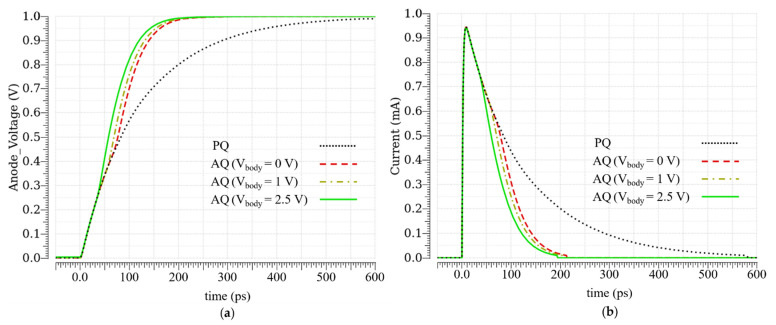
Post-layout simulation results in the PQ and the AQ: (**a**) anode voltage; (**b**) avalanche current.

In Figure 6a, the anode voltage in AQ (for different body voltages) and PQ, when an avalanche happens, is shown. Regarding the slope change in the anode voltage in the AQ (V_body_ = 2.5 V), the avalanche is surely detected in less than 40 ps, while the anode voltage is about 300 mV. This time is composed of the detection time and the time that P_2_ (AQ MOSFET) needs to reach its maximum current. From Figure 6a, it is clear that, in the AQ, the anode voltage reaches V_ex_ faster than in the PQ. 

Figure 6b shows the avalanche current in AQ and PQ. The avalanche in the AQ is quenched in about 200 ps, while it takes about 600 ps for the avalanche to be passively quenched. It can be observed that the area under the avalanche current curve in the AQ is less than this area in the PQ. In fact, this area is equal to the avalanche charge in the SCR.

**Figure 7:**
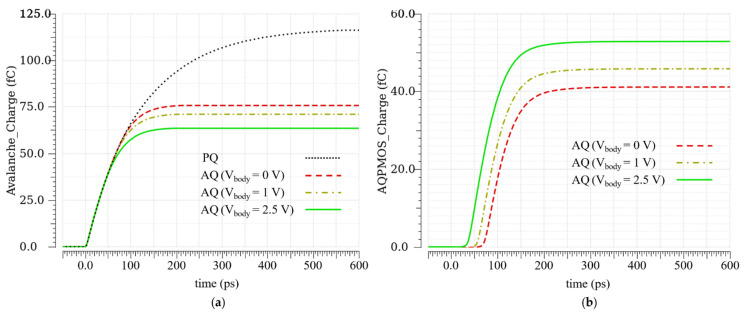
(**a**) Avalanche charge in the PQ and the AQ; (**b**) AQ MOSFET charge contribution during an avalanche.

Figure 7a is an integration of the curves in Figure 6b. It illustrates that the avalanche charge is reduced in the AQ in comparison with the PQ, and the amount of this charge reduction is exactly equal to the charge provided by the AQ MOSFET P_2_; see Figure 7b. Regarding Figure 7a, the avalanche charge in the PQ is about 114 fC, while, in the AQ (V_body_ = 2.5 V), this is only about 62 fC. The difference is equal to the charge provided by the P_2_ (V_body_ = 2.5 V), which is about 52 fC. Post-layout simulations prove that the proposed AQAR circuit reduces the avalanche charge up to 45%, which should also result in the same percentage of reduction for the afterpulsing.

### 4.2. Experimental Results

**Figure 8:**
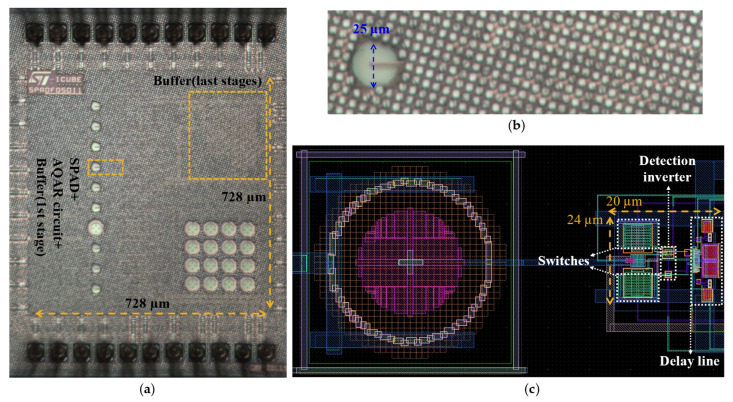
(**a**) Micrograph of the full chip; (**b**) the used pixel in this work; (**c**) layout view of the proposed pixel.

Figure 8a shows a micrograph of the chip. It includes different SPADs and different AQAR circuits, plus the controlling electronics. In addition, a wide-band (~3 GHz) analog buffer is designed and integrated in the chip to directly monitor the anode voltage variations at the moment of the avalanche. The internal area of the chip is 728 µm × 728 µm. The electronic of the pixel is hidden by the metal filling; therefore, a layout view is used in Figure 8c to illustrate the proposed AQAR circuit. The circuit is integrated with a circular SPAD with a diameter of 25 µm, which is reported in Reference [20]. The proposed circuit has a dimension of 20 µm × 24 µm, but most of this area is consumed by the two serial switches (P_3,_ N_3_), which are implanted for test purposes only. It is possible to integrate the P_1_ and the N_1_ in the same well to have a more compact layout; however, in this work, they have been placed in separate wells for testing purposes, as well.

**Figure 9:**
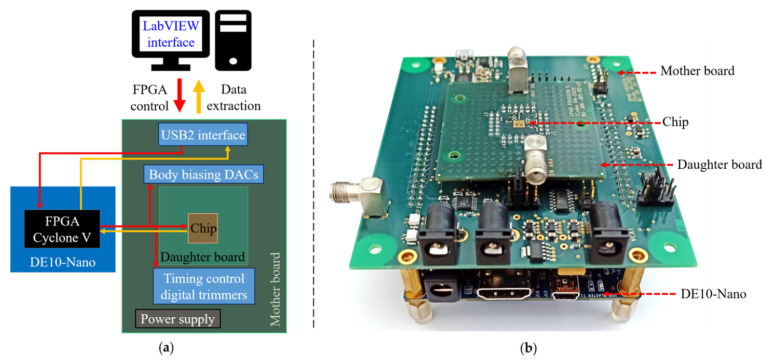
(**a**) Block diagram of the experimental setup; (**b**) photo of the experimental setup.

In order to characterize the prototype, a testing platform based on a field-programmable gate array (FPGA) DE10NANO board has been developed, as shown in Figure 9. The test chip was mounted on a daughter board which is plugged into a motherboard. The daughter board has an SMA connector that is connected to the output of the analog buffer to observe the anode voltage transient behavior. The motherboard integrates the power supplies of the chip, circuits for the configuration of the chip (i.e., a bit stream sent by the FPGA), digital to analog converters (DACs) to generate the body biasing voltages, digital trimmers to control the hold off and reset time of the circuit, and a USB2 controller for communication with a LabVIEW interface. The LabVIEW interface controls the system and reads out the data from the FPGA and allows saving them in a text file. The FPGA is configured from the LabVIEW interface to send configuration bits to the chip, to set the DAC and the digital trimmer of the motherboard. This bit stream, in particular, allows switching between PQ and AQ. The FPGA implantation also includes the afterpulsing measurement method, which consists of a 24 bits gray counter (operating at 200 MHz) written in an FIFO when a rising event occurs on the digital output of the AQAR circuit. This allows measuring the inter arrival time of the SPAD detected events with a resolution of 5 ns. All the test bench boards are powered by a laboratory power supply. The analog output is measured by a 13 GHz wide-band oscilloscope. And, finally, the testing platform (DE10NANO, daughter and motherboards) is placed in an environmental chamber for constant temperature and hygrometry operating condition. All the results are based on the DCR measurements at 40 °C, while the SPAD is set in the free-running mode in the dark.

**Figure 10:**
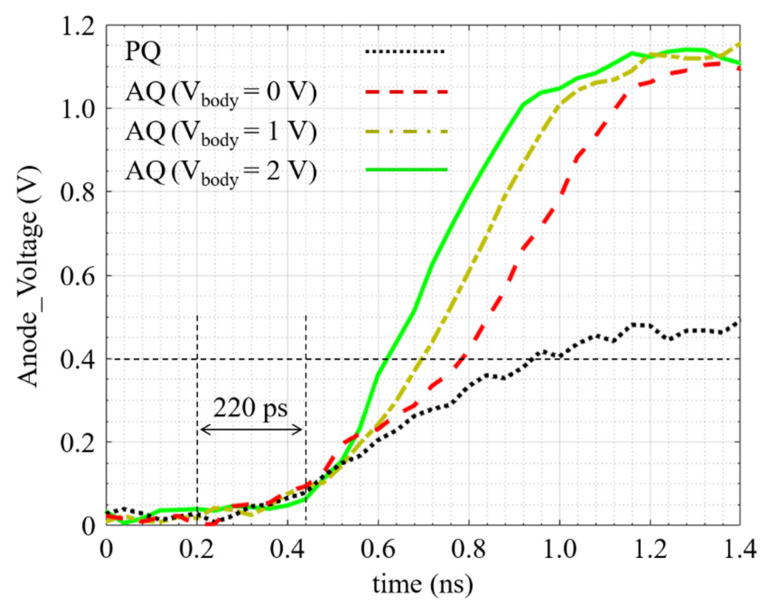
Anode voltage transient in the PQ and the AQ when an avalanche occurs. The excess bias voltage is about 400 mV.

Figure 10 is the experimental replica of Figure 6a. Thanks to the integrated wide-band analog buffer, the anode voltage is measured when an avalanche occurs. By changing the body voltage through the DAC, different anode voltage curves are extracted for V_body_ = 0, 1, and 2 Volts. For a higher body voltage, some spurious avalanches are detected by the AQ circuit, indicating that the threshold is too low, and the circuit triggers with the noise. In addition, due to the SPAD noise, the excess bias voltage is limited to about 500 mV as, above this value, the SPAD is saturated. Figure 10 shows how much faster the proposed AQ circuit biases the SPAD out of the breakdown region in comparison with the PQ. For instance, the anode voltage curve in AQ with V_body_ = 2 V passes the 400 mV excess bias 300 ps sooner than the PQ curve. Regarding the slope change in the AQ curve with V_body_ = 2 V, the avalanche is detected in about 220 ps. The difference between this time in the simulation results and in the experimental results is because the SPAD model used in the simulation is much faster than the actual integrated SPAD.

**Figure 11:**
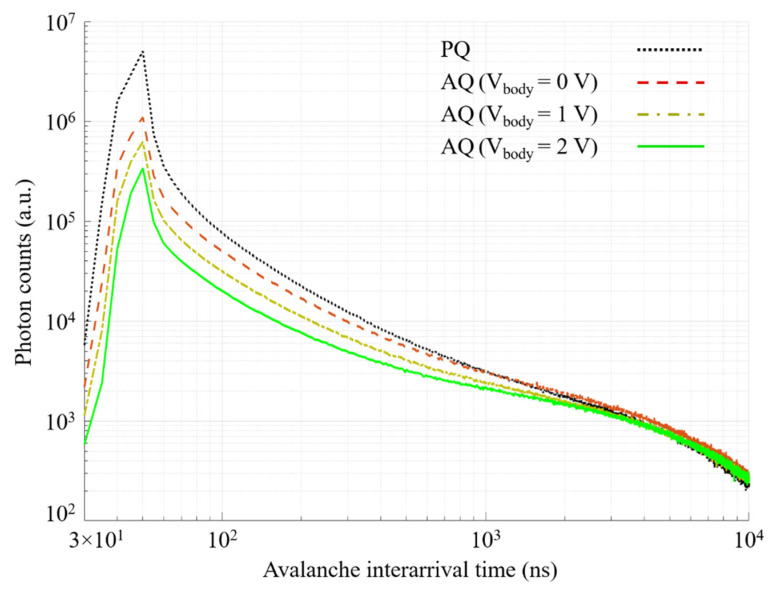
Avalanche interarrival time histogram in the PQ and the AQ for a hold off time of 5 ns. The excess bias voltage is 400 mV.

Figure 11 shows the avalanche interarrival time histogram in PQ and AQ with different body voltages. It is worth mentioning that, in order to have a fair comparison between the PQ and the AQ circuits, the reset is always set to the active mode after a hold off time set to only 5 ns. Thanks to the gray counter, the intervals between two consecutive avalanches are calculated to obtain the histogram of the interarrival time. Since the probability of the afterpulsing is very low after a few microseconds, this part of the histogram (in our case, after 6 µs) is fitted by an exponential function to obtain the afterpulsing-free DCR curve, modeled as a Poisson process. Then, for an interarrival time of less than 10 µs (to guarantee that no afterpulse is missed), the afterpulsing effect is extracted as the area between the measured interarrival time histogram and the fitted afterpulsing-free DCR curve. By subtracting the fitted DCR curve from the interarrival time histogram, the number of afterpulses during the measurement is extracted. It is clearly observable in Figure 11 that the higher the body voltage, the lower the detection threshold, and so the lower the afterpulsing effect.

**Figure 12:**
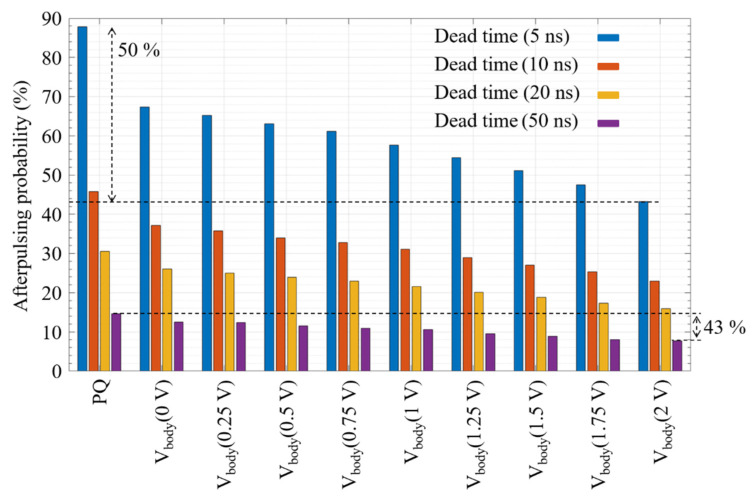
Afterpulsing probability in the PQ and the AQ with different body voltages for hold-off times of 5, 10, 20, and 50 ns. The excess bias voltage is 400 mV.

The same procedure is performed for different dead times, and the results are announced in Afterpulsing Probability Percentage (APP) (the ratio between the counted number of afterpulses and the total count of the histogram). Figure 12 shows the afterpulsing probability for hold off times of 5 ns, 10 ns, 20 ns, and 50 ns, while the body voltage is swept from 0 V to 2 V to investigate the effectiveness of the proposed circuit in the afterpulsing reduction regarding the PQ. In 5 ns, 10 ns, and 20 ns of the dead time, the afterpulsing reduces by a factor of 2 (50%), while, in the 50 ns, the reduction is slightly less (43%). This is due to this fact that, after 50 ns, some of the carriers are already released from the trapping centers. It is obvious that, with a hold off time of more than 10 µs, the efficiency of the AQ circuit becomes negligible, as the afterpulsing effect itself is also negligible. 

Even though the absolute value of the afterpulsing is high due to the used SPAD design, the relative reduction of the afterpulsing effect of the proposed AQ circuit should remain the same with a less noisy SPAD. These results justify that the proposed AQAR circuit alleviate the common tradeoff between the afterpulsing and the dynamic range of the SPAD by enabling the increase of the maximum photon count rate, thanks to the afterpulsing reduction by a factor of two at a very short hold off time. 

Several reported works have aimed APP reduction. A few of them have clearly quantified the afterpulsing reduction in comparison with PQ [7,15,16,21,22]. However, it is hard to do a fair comparison between our work and the other state-of-the-art works as most of them have been done in the compound semiconductor technologies that specifically have been adapted for the SPAD applications or CMOS technologies with a much higher voltage headroom than the technology used in our work. 

**Figure 13:**
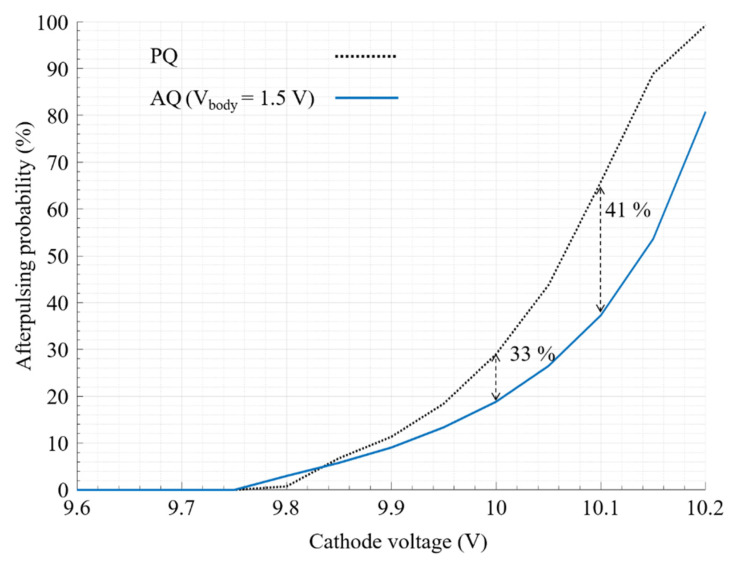
Afterpulsing probability versus excess bias voltage in the PQ and the AQ with 1.5 V of body voltage. The hold of time is 20 ns.

A comparison between APP in PQ and AQ is made, while the excess bias voltage (cathode voltage) is increasing. The body voltage is 1.5 V, and the hold off time is set to 20 ns; see Figure 13. The true breakdown voltage of the used SPAD is actually 9.6 V, in which we start to observe some very weak avalanches (low amplitude anode voltage signal), thanks to the analog buffer. In fact, the amplitude of the avalanches is lower than the threshold of the readout electronics; thus, they cannot be detected. This is why, in Figure 13, the first value of APP is recorded in about 9.75 mV. Another interesting point in Figure 13 is that the APP of AQ is higher than the one of PQ until 9.84 mV. Yet, the reason is that the anode voltage amplitude is not very high; therefore, some of them are missed in the PQ. On the contrary, in the AQ, when these very weak avalanches are detected by the body biased inverter, the AQ PMOS (P_2_) raises their amplitude, so they can be detected by the following digital gate. By increasing the excess bias voltage, the AQAR circuit has more room to contribute in the quenching process, so, as it is shown in Figure 13, the afterpulsing reduction is increasing when the excess bias voltage is increased, except for a cathode voltage above 10.15 V. Indeed, as the SPAD in PQ mode is saturated for a cathode voltage above 10.15 V, where the afterpulsing effect is also saturating at 100%, the afterpulsing relative reduction appears to be lowered.

## 5. Conclusions

In this paper, a body biased, inverter base AQAR circuit is presented to reduce the afterpulsing effect in a 28 nm FD-SOI CMOS technology. The body biasing technique, along with a proper sizing, are used to design a very fast and low threshold inverter to detect the avalanche. The detection inverter is able to detect the avalanche faster than 40 ps, while the avalanche is at its very beginning. This detection results in the avalanche charge reduction, thanks to an AQ MOSFET. It is shown that the amount of the avalanche charge reduction is equal to the amount of the charge contribution of the AQ MOSFET. Experimental results prove that the avalanche charge reduction is translated to the afterpulsing reduction. We illustrated how tuning the body voltage of the detection inverter controls the effectiveness of the AQAR circuit. For a body voltage of 2 V, the afterpulsing is reduced by 50%, with a dead time of only 5 ns. Thus, the proposed circuit alleviates the tradeoff between the afterpulsing and the dynamic range of the SPAD.

## Data Availability

Not applicable.
